# Non-linear effects and thermoelectric efficiency of quantum dot-based single-electron transistors

**DOI:** 10.1038/s41598-017-14009-4

**Published:** 2017-11-01

**Authors:** Vincent Talbo, Jérôme Saint-Martin, Sylvie Retailleau, Philippe Dollfus

**Affiliations:** 1 Univ. Grenoble Alpes, CEA, INAC-Pheliqs, 38000 Grenoble, France; 2 Centre de Nanosciences et de Nanotechnologies, CNRS, Univ. Paris-Sud, Université Paris-Saclay, C2N – Orsay, 91405 Orsay, cedex France

## Abstract

By means of advanced numerical simulation, the thermoelectric properties of a Si-quantum dot-based single-electron transistor operating in sequential tunneling regime are investigated in terms of figure of merit, efficiency and power. By taking into account the phonon-induced collisional broadening of energy levels in the quantum dot, both heat and electrical currents are computed in a voltage range beyond the linear response. Using our homemade code consisting in a 3D Poisson-Schrödinger solver and the resolution of the Master equation, the Seebeck coefficient at low bias voltage appears to be material independent and nearly independent on the level broadening, which makes this device promising for metrology applications as a nanoscale standard of Seebeck coefficient. Besides, at higher voltage bias, the non-linear characteristics of the heat current are shown to be related to the multi-level effects. Finally, when considering only the electronic contribution to the thermal conductance, the single-electron transistor operating in generator regime is shown to exhibit very good efficiency at maximum power.

## Introduction

Thermoelectric effects refer to the ability of a material to directly convert internal heat fluxes into electrical power and vice-versa, via the so-called Seebeck and Peltier effects, respectively^1^. In the linear regime, the maximum efficiency of the thermoelectric (TE) conversion is expressed as a function of the unitless TE figure of merit defined as $ZT={G}_{e}{\alpha }^{2}T/K$ where *G*_*e*_ is the electronic conductance, *α* the Seebeck coefficient, and *K* the thermal conductance^2^. To achieve high efficiency of TE conversion, high *ZT* values are desirable, but the common *ZT* values available today in TE materials are limited to about 1, which leads to weak efficiency with respect to standard heat engines^3^. Indeed, TE properties of bulk materials suffer from strong interdependence between the key parameters. The electronic and thermal conductivities are linked via the Wiedemann-Franz law, while according to the Mott formula the electronic conductivity and the Seebeck coefficient have opposite behaviors when approaching the Fermi level.

Research activities on thermoelectrics have been the subject of a renewed interest in the last two decades^4–6^ thanks to both the recent progress in nanotechnology and the pioneering publications of Hicks and Dresselhaus^7,8^ that pointed out that structures with reduced dimensionality exhibit better TE efficiency than their bulk counterpart. Two strategies were used to improve *ZT*. The first strategy focuses on the denominator of *ZT* and targets at increasing phonon scattering, in particular at the nanostructure boundaries, to reduce the lattice thermal conductance. The second strategy aims at improving the power factor ${G}_{e}{\alpha }^{2}$ thanks to bandgap broadening and the enhancement resulting from the nonlinearities induced in the electronic density-of-states (DOS) of low dimensional systems^9^. Many theoretical and experimental efforts have been performed to investigate thermoelectric properties of nanostructures such as superlattices^10,11^, graphene nanostructures^12^, semiconducting nanowires with various sizes and doping levels^13,14^.

If Silicon is prominent in microelectronics, Tellurium alloys as PbTe and Bi_2_ Te_3_ are dominating the TE market along with some more complex alloys^5^. TE properties of Silicon and SiGe have also been investigated^4,14^ and nanowires exhibiting ultra-low thermal conductivities appear to be particularly relevant^13,15^ in the context of TE applications.

Ultimately, in zero-dimensional (0D) structures, the delta-shape density of states could lead to the “best thermoelectric material”^16^. In such an “ideal” system of discrete levels, i.e. with negligible broadening, the efficiency tends to the maximum Carnot efficiency *η* = 1 − *T*_*c*_/*T*_*h*_, where *T*_*h*_ and *T*_*c*_ are the hot (h) and a cold (c) temperatures, respectively^17,18^.

The efficiency at maximum power of resonant-tunneling quantum dot (QD) structures was investigated recently^19–21^. Some early works on QD-based structures have shown the typical sawtooth-like oscillations of the Seebeck coefficient as a function of gate bias^22,23^, and one of the first *ZT* value higher than one was measured in a QD superlattice^24^. Since then, a tremendous amount of theoretical^25,26^ and experimental^27–29^ works on QDs as energy harvesters has been produced, as reviewed by Sothmann, Sanchez and Jordan^30^. With the advent of TE studies in nanostructures, non-linearity effects have been evidenced and widely studied in the recent years^31–37^, as reviewed recently by Benenti *et al*.^38^.

Among QD-based devices, a promising candidate for TE applications is the single-electron transistor (SET)^39–42^, initially introduced by Averin and Likarev^43,44^. The discreteness of its DOS provides in principle a fine control of current thanks to the Coulomb blockade effect that reduces thermal losses. Taking advantage of advances in the reduction of QD sizes down to a few nanometers, Silicon-based SETs can now operate at room-temperature, demonstrating nice Coulomb oscillations^45,46^.

In this work, the thermoelectric efficiency and the electric power of a Silicon QD-based SET operating around 100 K is simulated, beyond the linear response regime, where the figure of merit can no longer be directly linked to thermal efficiency, thus requiring its calculation. The main originality of the present study is to focus on a sequential transport regime including the energy level broadening induced by electron-phonon scattering. Even if most of TE studies in QDs are made in the framework of resonant tunneling transport, i.e. within the Landauer formalism, our assumption of a sequential tunneling regime is justified by the fact that electron-phonon scattering rates in the simulated QD are greater than tunneling rates^47^. The impact of sequential tunneling in QDs on thermopower has been studied previously by Scheibner *et al*. for temperatures lower than 1.5 K^48^. Here, we intend to describe properly at finite temperature both quantum confinement and Coulombic effects occurring in a semiconducting QD, through the fully self-consistent solution of Poisson’s and Schrödinger’s equations.

On this purpose, we used our home-made 3D simulator SENS (Single-Electron Nanostructure Simulation)^47,49,50^ dedicated to QD-based single-electron devices, which is well suited for investigating TE properties as it allows us to describe the electrical behavior of such devices at any finite temperature^51^. It should be emphasized that this work focuses on the intrinsic thermoelectric behaviour of the electronic system, in the absence of phonon-mediated heat flow from source to drain contacts. The latter can be added as an additional external contribution, which will be discussed. Details about the model are given in the second section. The thermoelectric figure of merit, power and efficiency of a given SET are presented and discussed in the third section as a function of the strength of electron-phonon coupling. In particular, the non-linear effects that become relevant under large bias voltage are investigated.

## Model

The code SENS has been used previously to investigate different types of QD-based single-electron devices, such as double^49^ and triple^47^ tunnel junctions, and has been extended to the case of the SET^50^. The simulation of steady-state electrical and thermal device characteristics relies on three stages. First, a 3D solver of coupled Poisson’s and Schrödinger’s equations provides the electronic structure of the QD according to the number of particles it contains. Then, the tunnel transfer rates are calculated through the Fermi golden rule within the Bardeen’s formalism, and finally, these rates are introduced into the Master equation to calculate the charge and heat currents.

### Electronic structure of Si-QDs

In this approach, the electronic structure of the QD is calculated through the self-consistent solution of the 3D Poisson’s and Schrödinger’s equations, within the effective mass and Hartree approximations, which have been proven to be correct for Si QDs of diameter greater than 3 nm^52,53^. The advantage of the Hartree method is that it gives access to the wave-functions *ψ*_*i*_ and the energy level *E*_*i*_ for each electron *i* in the QD. This is very convenient for the calculation of tunnel transfer rates. For the *i*-th electron among a number *n* of electrons in the QD and for a given drain and gate bias configuration, the Poisson-Schrödinger system of equations to be solved is

1 $$\{\begin{array}{l}[-\frac{{\hslash }^{2}}{2}\overrightarrow{\nabla }(\frac{1}{m}\overrightarrow{\nabla })+{V}^{conf}(\overrightarrow{r})+{V}^{pol}(\overrightarrow{r})+{V}_{i}^{coul}(\overrightarrow{r})]{\psi }_{i}(\overrightarrow{r})={E}_{i}{\psi }_{i}(\overrightarrow{r})\\ \overrightarrow{\nabla }(\varepsilon {\varepsilon }_{0}\overrightarrow{\nabla }{V}_{i}^{coul}(\overrightarrow{r}))=\bar{e}{\rho }_{i}(\overrightarrow{r})\end{array}$$

where $\bar{e}$ is the single electron charge, *m* an isotropic effective mass,*ε* is the dielectric constant, *V* ^*conf*^ is the confinement potential and *V* ^*pol*^ is the bias potential, obtained by solving the 3D Poisson equation without charge. The Coulomb potential ${V}_{i}^{coul}$ for the *i*-th electron is obtained from the charge density *p*_*i*_ given by

2 $${\rho }_{i}(\overrightarrow{r})=\bar{e}\sum _{i\ne j}{|{\psi }_{i}(\overrightarrow{r})|}^{2}$$

In this simple model of Si conduction band with isotropic effective mass, each energy level is twelve-fold degenerate, i.e. with a factor of 2 for the spin degeneracy and a factor of 6 for the valley degeneracy. In this work the range of SET operation is such that the number of electrons in the dot is not higher than 2. It is thus a single-level system with an energy level depending on the number *n* of electrons in the dot. Most quantities are thus dependent on this number *n* that is used below either as a variable or as a label for each quantity related to the energy level.

### Tunnel transfer rates

We assume that the gate oxide is thick enough for the tunneling between the QD and the gate to be negligible. Then the tunnel transfer rates between the source or drain lead L and the dot d are calculated using the Fermi golden rule, i.e. in the weak coupling limit. In the case of discrete energy levels of negligible width, the transfer rates ${{\rm{\Gamma }}}_{Ld}$ (lead-to-dot) and Γ_*dL*_ (dot-to-lead) are thus given by

3 $$\{\begin{array}{ll}{{\rm{\Gamma }}}_{Ld}(n,{T}_{L}) & =\frac{2\pi }{\hslash }|{M}_{n+1}{|}^{2}{\rho }_{L}{l}_{{\mu }_{n}}{f}_{L}({\mu }_{n+1},{T}_{L})\\ {{\rm{\Gamma }}}_{dL}(n,{T}_{L}) & =\frac{2\pi }{\hslash }|{M}_{n}{|}^{2}{\rho }_{L}{g}_{{\mu }_{n}}[1-{f}_{L}({\mu }_{n},{T}_{L})]\end{array}$$

where *n* is the number of electron in the dot before tunneling, ${g}_{{\mu }_{n}}$ is the number of electrons on the energy level *μ*_*n*_, ${l}_{{\mu }_{n}}$ the number of free states on this level, *T*_*L*_ the temperature of the lead, *f*_*L*_(*μ*_*n*_,*T*_*L*_) the Fermi-Dirac distribution function at lead Fermi level ${E}_{{F}_{L}}$, and *ρ*_*L*_ the density of states of the lead. The tunneling matrix element *M*_*n*_ is given by the Bardeen formula^54,55^

4 $${M}_{n}=\frac{{\hslash }^{2}}{2{m}_{barr}}{\iint }_{{S}_{barr}}[{\psi }_{L}(\overrightarrow{r})\overrightarrow{\nabla }{\psi }_{d,n}(\overrightarrow{r})-{\psi }_{d,n}(\overrightarrow{r})\overrightarrow{\nabla }{\psi }_{L}(\overrightarrow{r})]d\overrightarrow{S}$$

where *S*_*barr*_ is a surface inside the tunnel barrier which separates arbitrarily the dot domain from the electrode domain^55,56^, ${\psi }_{d,n}(\overrightarrow{r})$ and ${\psi }_{L}(\overrightarrow{r})$ are the electronic wave functions in the QD and the lead, respectively, and *m*_*barr*_ is the electron effective mass in the tunnel barrier. The wave function *ψ*_*L*_ is deduced from an analytical expression derived within the Wentzel-Kramers-Brillouin (WKB) approximation, which has been proven correct by comparing the tunneling currents obtained for the simple case of a Gold/SiO2/Gold structure using both our approach and an exact calculation^55^.

### Electric current

**Figure 1:**
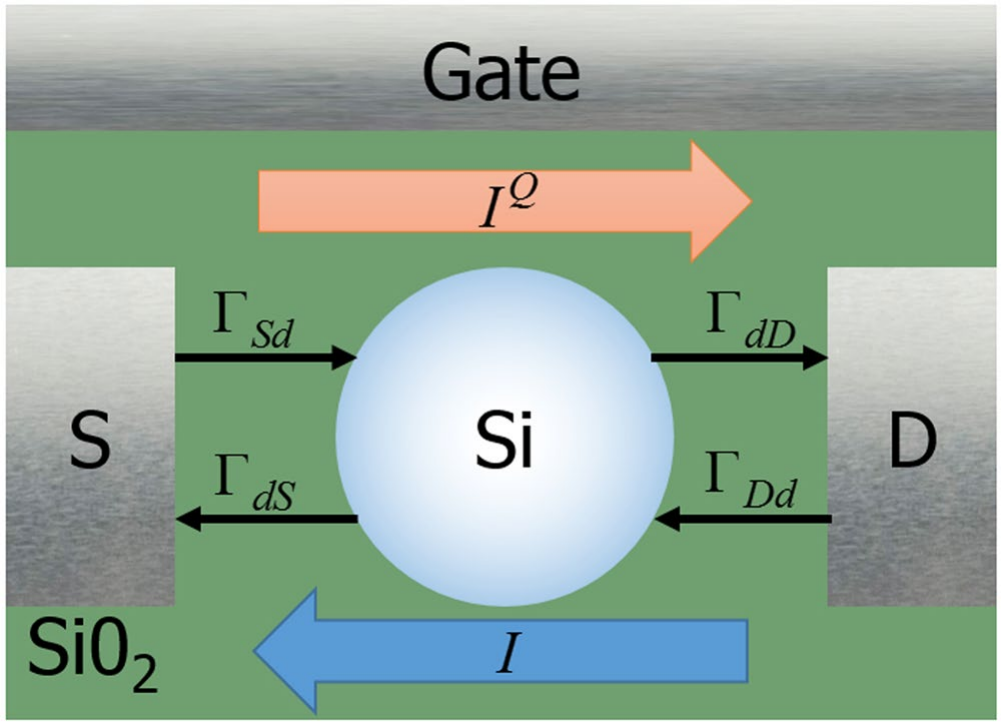
Schematic view of the SET investigated in this work. Arrows are displayed to indicate the sign convention for currents and transfer rates.

The tunnel transfer rates are then introduced into the Master equation to deduce the probabilities *P*(*n*) of finding *n* electrons in the dot. Using the sign convention of Fig. 1, the electronic current *I* as well as the heat currents ${I}_{S}^{Q}$ and ${I}_{D}^{Q}$, are given by

5 $$I={I}_{S}={I}_{D}=e\sum _{n}P(n)[{{\rm{\Gamma }}}_{Sd}(n,{T}_{S})-{{\rm{\Gamma }}}_{dS}(n,{T}_{S})]=e\sum _{n}P(n)[{{\rm{\Gamma }}}_{dD}(n,{T}_{D})-{{\rm{\Gamma }}}_{Dd}(n,{T}_{D})]$$

6 $${I}_{S}^{Q}=\sum _{n}({\mu }_{n}-{E}_{{F}_{S}})[P(n-\mathrm{1)}{{\rm{\Gamma }}}_{Sd}(n-\mathrm{1,}\,{T}_{S})-P(n){{\rm{\Gamma }}}_{dS}(n,\,{T}_{S})]$$

7 $${I}_{D}^{Q}=\sum _{n}({\mu }_{n}-{E}_{{F}_{D}})[P(n){{\rm{\Gamma }}}_{dD}(n,{T}_{D})-P(n\,-\,\mathrm{1)}{{\rm{\Gamma }}}_{Dd}(n\,-\,\mathrm{1,}{T}_{D})]$$

where $({\mu }_{n}\,-\,{E}_{{F}_{L}})$ is the energy extracted from the lead reservoir when the transfer occurs. However, in order to describe more realistic systems, one must consider the broadening of energy levels due to phonon coupling.

### Broadening of energy levels and heat current

Since tunneling between dot and electrodes is considered as sequential, electrons are assumed to rapidly lose their quantum coherence in the QD due to interactions in the dot, and in particular to electron-phonon coupling. The consequence of these interactions is a broadening of energy levels, called collisional broadening^57^, which is not negligible in Si-QDs^57–61^. They have been implemented previously in this code for the simulation of double-dot structures^47^. It should be noted that in this device working in sequential regime, with tunneling rates smaller than 4 kHz, the broadening induced by the weak coupling to source and drain is negligible compared to that due to the electron-phonon coupling.

To take into account the broadening of levels, we consider a spectral function *A*(*ε*) representing the density of probability that the electron in the dot has an energy between *E* and *E* + *dE*. The expression of the spectral function defined in^47^ is complex and depends on both the temperature and the frequency of the phonon involved. Using a Monte-Carlo algorithm, it is possible to accurately consider the phonon frequency-dependence of spectral function^47^, which leads to large computation times. Here, we make the approximation of a single Lorentzian function centered on the equilibrium energies *μ*_*n*_, commonly used in the frame of the Landauer formalism for coherent transport^62,63^, i.e.

8 $$A(\varepsilon )=\frac{{(H\mathrm{/2)}}^{2}}{{\varepsilon }^{2}-\,{(H\mathrm{/2)}}^{2}}$$

where *ε* = *E* − *μ*_*n*_ is the energy deviation from the discrete energy level *μ*_*n*_ and *H* is the full width at half maximum (FWHM) of the spectral function. With the introduction of level broadening, the expressions for tunnel transfer rates (3) become

9 $$\{\begin{array}{ll}{\Gamma }_{Ld}(n,{T}_{L},\varepsilon ) & =\frac{1}{\hslash }|{M}_{n+1}{|}^{2}{\rho }_{L}({\mu }_{n+1}+\varepsilon ){l}_{{\mu }_{n}}{f}_{L}({\mu }_{n+1}+\varepsilon ,{T}_{L})A(\varepsilon )\\ {\Gamma }_{dL}(n,{T}_{L},\varepsilon ) & =\frac{1}{\hslash }|{M}_{n}{|}^{2}{\rho }_{L}({\mu }_{n}+\varepsilon ){g}_{{\mu }_{n}}[1-{f}_{L}({\mu }_{n}+\varepsilon ,{T}_{L})]A(\varepsilon )\end{array}$$

From these tunnel rates, we determine the probabilities *P*_*ε*_(*n*) to find *n* electrons in the dot with a deviation *ε* from equilibrium, and the expressions for currents (5–7) are now given by

10 $$I={\int }_{\varepsilon }\sum _{n}e{P}_{\varepsilon }(n)[{{\rm{\Gamma }}}_{Sd}(n,{T}_{S},\varepsilon )-{{\rm{\Gamma }}}_{dS}(n,{T}_{S},\varepsilon )]$$

11 $${I}_{S}^{Q}={\int }_{\varepsilon }\sum _{n}({\mu }_{n}+\varepsilon -{E}_{{F}_{S}})[{P}_{\varepsilon }(n-\mathrm{1)}{{\rm{\Gamma }}}_{Sd}(n-\mathrm{1,}{T}_{S},\varepsilon )-{P}_{\varepsilon }(n){{\rm{\Gamma }}}_{dS}(n,{T}_{S},\varepsilon )]$$

12 $${I}_{D}^{Q}={\int }_{\varepsilon }\sum _{n}({\mu }_{n}+\varepsilon -{E}_{{F}_{D}})[{P}_{\varepsilon }(n){{\rm{\Gamma }}}_{dD}(n,{T}_{D},\varepsilon )-{P}_{\varepsilon }(n-\mathrm{1)}{{\rm{\Gamma }}}_{Dd}(n-\mathrm{1,}{T}_{D},\varepsilon )]$$

### Thermoelectric coefficients

All data required to calculate the figure of merit can be obtained from the expression of electronic and heat currents, except the part of thermal conductance contributed by phonons themselves that is not calculated in our simulation. In the linear regime, and assuming that source and drain are connected to heat reservoirs of temperature *T*_*h*_ and *T*_*c*_, respectively, the Onsager-Callen formalism defines the electronic and heat currents as (with sign convention of Fig. 1)^64,65^

13 $$I=\alpha {G}_{e}{\rm{\Delta }}T+{G}_{e}{V}_{DS}$$

14 $${I}^{Q}=\alpha IT+K{\rm{\Delta }}T$$

where Δ*T* = *T*_*h*_ − *T*_*c*_ and *T* = (*T*_*h*_ + *T*_*c*_)/2. The term *αIT* is the advective contribution (thermal transfer linked with a macroscopic transport) while −*K*Δ*T* corresponds to the conductive term, i.e, the thermal loss. We can extract from simulations the parameters

15 $${G}_{e}={(\frac{dI}{d{V}_{DS}})}_{{\rm{\Delta }}T=0}$$

16 $$\alpha =\frac{1}{{G}_{e}}{(\frac{dI}{d{\rm{\Delta }}T})}_{{V}_{DS}=0}=-{(\frac{{V}_{DS}}{{\rm{\Delta }}T})}_{I=0}$$

17 $$K={(\frac{{I}^{Q}}{{\rm{\Delta }}T})}_{I=0}$$

## Results and Discussion

### Studied SET

The simulated SET consists in a spherical Si-QD of 4.4-nm diameter with 1.2-nm (1.5-nm)-thick source (drain) tunnel barriers and a 5-nm-thick gate oxide. These tunnel barriers are thin here to achieve relatively high electronic conductance. Since a finite mean temperature *T* = 100 K was considered, the broadening of energy levels induced by electron-phonon scattering must be included. According to the rigorous calculations by Valentin *et al*.^47^, the most likely phonon modes activated in such structure at this temperature correspond to spectral functions with FWHMs ranging from H = 0.001*k*_*B*_ *T* to 0.05*k*_*B*_ *T*. In this study, the case of discrete energy levels is considered as a reference to be compared with the results obtained in the case of Lorentzian broadening of energy levels with aforementioned realistic FWHMs. For comparison, the results obtained for an unrealistically-wide broadening of H = *k*_*B*_ *T* were also considered.

To investigate the thermoelectric behavior of the SET, a temperature gradient Δ*T* was applied by fixing the temperature of the source and drain reservoirs at *T* + Δ*T*/2 and *T* − Δ*T*/2, respectively, with Δ*T* ranging from 0 to 10 K.

### I-V characteristics

**Figure 2:**
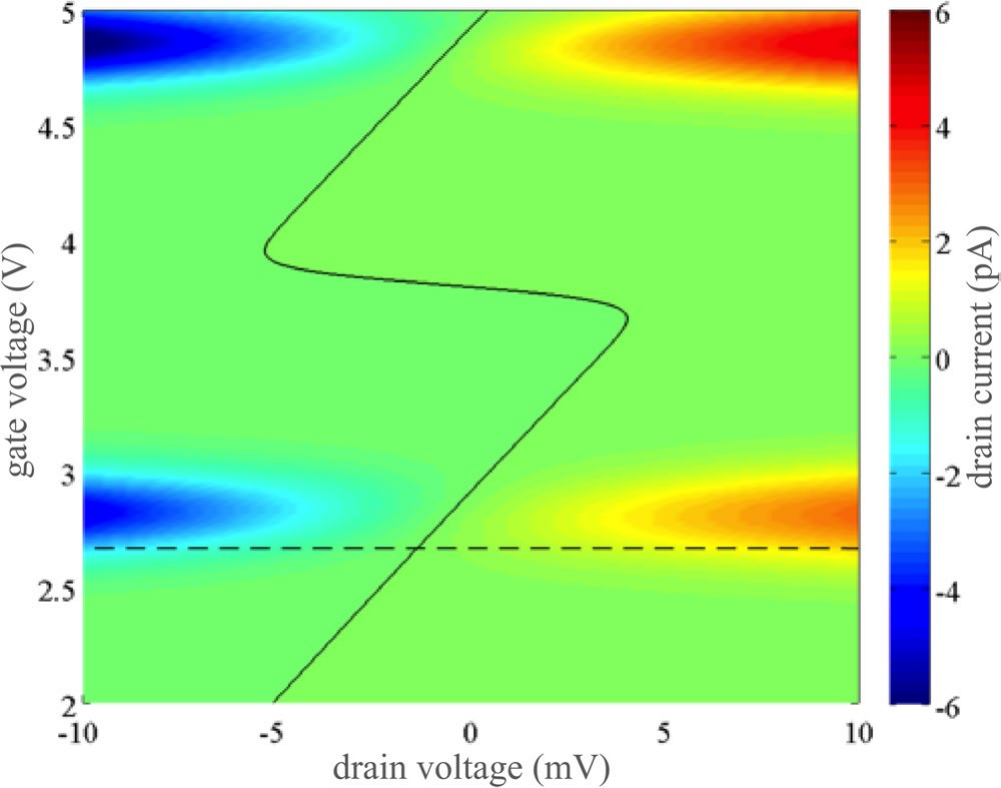
Drain current stability diagram for *T* = 100 K and Δ*T* = 5 K. The solid line corresponds to the current value *I* = 0, the dashed line to *V*_*GS*_ = 2.67 V.

**Figure 3:**
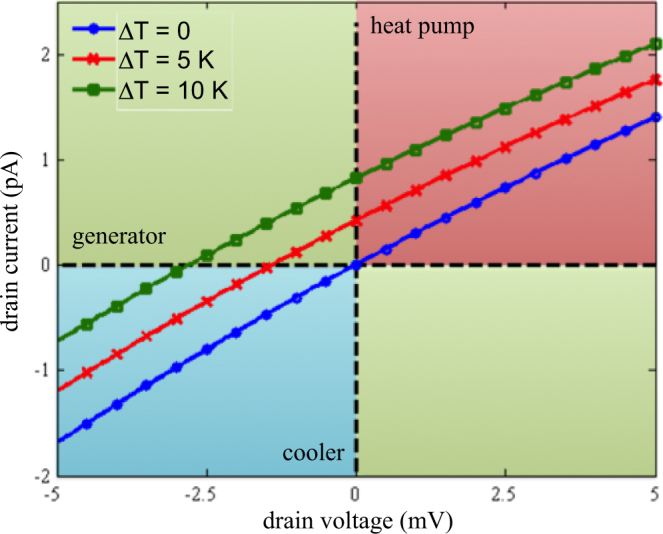
Current-drain voltage characteristics for three temperature gradients Δ*T* at *V*_*GS*_ = 2.67 V.

The drain current stability diagram is plotted in Fig. 2 with zooming in on small drain bias regime. The center of typical Coulomb diamonds, commonly observed in experimental SETs, corresponds to the Coulomb blockade zones where *I* ≈ 0. To use this SET as a thermoelectric generator, it is necessary to select a gate voltage *V*_*GS*_ for which the Coulomb blockade vanishes, i.e. the conductance is finite, when applying a small drain voltage. This behavior is achieved for *V*_*GS*_ around 2.67 V (horizontal dashed line in Fig. 2). At this gate bias the *I* − *V*_*DS*_ characteristics, plotted in Fig. 3, are quasi-linear at small drain voltage in the mV range and the electronic conductance *G*_*e*_ is almost constant.

When a positive temperature gradient Δ*T* is applied the *I* − *V*_*DS*_ characteristic shifts upwards, which is the signature of a thermoelectric effect. According to Eqs. (3), the temperature of electrodes influences the tunnel transfer rates via the Fermi-Dirac distributions. Since at *V*_*GS*_ = 2.67 V we have ${E}_{{F}_{L}} < {\mu }_{1}$, for Δ*T* > 0 we have ${f}_{L}(T+{\rm{\Delta }}T\mathrm{/2)} > {f}_{L}(T) > {f}_{L}(T-{\rm{\Delta }}T\mathrm{/2)}$, thus ${{\rm{\Gamma }}}_{Sd}(T+{\rm{\Delta }}T\mathrm{/2)} > {{\rm{\Gamma }}}_{Sd}(T)$ and ${{\rm{\Gamma }}}_{dD}(T-{\rm{\Delta }}T\mathrm{/2)} > {{\rm{\Gamma }}}_{dD}(T)$. As both source-to-dot and dot-to-drain tunnel transfer rates increase with *V*_*DS*_, the drain current increases with a positive temperature gradient Δ*T*, and a finite current takes place at *V*_*DS*_ = 0 V.

Different working regimes can be observed. In the regimes of heat pump (*V*_*DS*_ > 0 and *I* > 0) and cooler (*V*_*DS*_ < 0 and *I* < 0) the QD is a passive system that uses an electrical command to control the thermal flux exchanged between the thermal reservoirs (or contacts). In the third regime (*V*_*DS*_ < 0 and *I* < 0), the QD acts as a generator that can harvest a part of the existing thermal flux into useful electrical power. The drain voltage for which the current is null is usually called the “open-circuit” voltage and is denoted as *V*_*OC*_ in what follows.

For a given temperature gradient and a given gate bias, both the Seebeck coefficient *α* (via (16)) and electronic conductance Ge (via (15)) can be extracted from the *I* − *V*_*DS*_ curves shown in Fig. 3.

### Seebeck coefficient and broadening of energy levels

**Figure 4:**
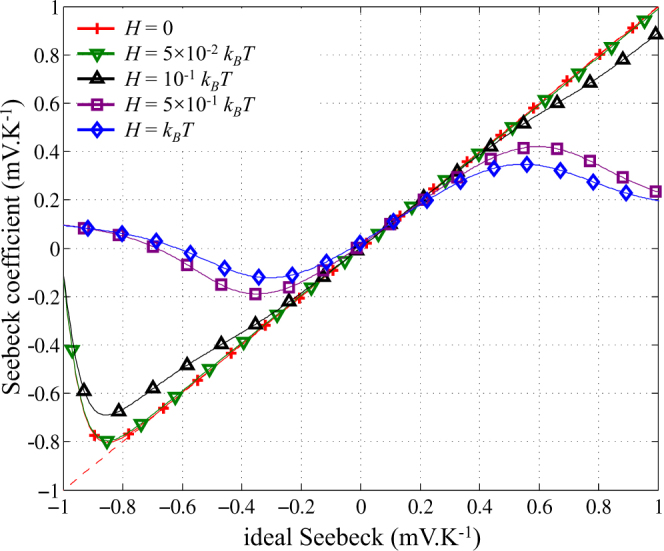
Seebeck coefficient as a function of “ideal” Seebeck coefficient $({\mu }_{n}-{E}_{{F}_{L}})/eT$ for different values of the energy of level broadening *H*. *T* = 100 K.

The Seebeck coefficient *α* is plotted in Fig. 4 as a function of $({\mu }_{n}-{E}_{{F}_{L}})/eT$ for different widths *H* of energy level broadening. If non-broadened energy levels are considered, *α* acts as an ideal Seebeck coefficient as described in^66^, i.e. $\alpha =({\mu }_{n}-{E}_{{F}_{L}})/eT$, except in the [−1*V* − 0.8 *V*] range, due to the transition between the states with one and two electrons in the QD, while the ideal Seebeck expression is valid only for a single-level QD. The linear behavior is preserved around the energy levels even if a large energy broadening up to *H* = 5 × 10 ^−^^2^ *k*_*B*_ *T* is considered. Since only unrealistic broadenings higher than *H* = 0.1*k*_*B*_ *T* have a significant impact, this linear behavior of the Seebeck coefficient appears to be highly robust to perturbations. As the relationship between *μ*_*n*_ and the gate voltage is only governed by the gate capacitance which can be accurately evaluated separately, the Seebeck coefficient in a SET could find some interesting applications in the field of metrology as a nanoscale standard for the Seebeck effect^51,66^.

### Seebeck coefficient and power factor

**Figure 5:**
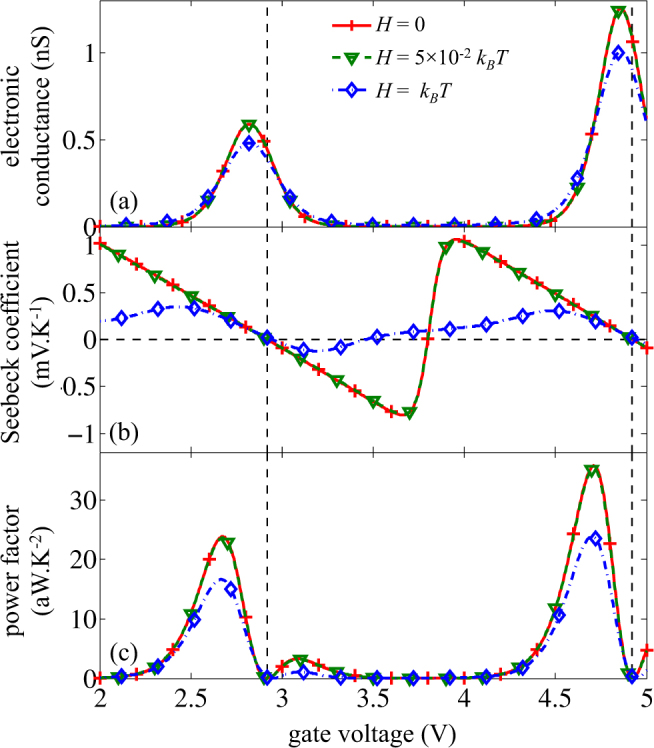
(**a**) Electronic conductance, (**b**) Seebeck coefficient and (**c**) power factor as a function of the gate bias for non-broadened and broadened energy levels with *H* = 0.05*k*_*B*_ *T* and *H* = *k*_*B*_ *T*. Vertical dotted lines correspond to ${E}_{{F}_{L}}=\mu$.

The electrical conductance, the Seebeck coefficient and the power factor calculated with and without energy broadening are presented in Fig. 5. A realistic broadening value lower than *H* = 0.05*k*_*B*_ *T* has no effect on the plotted parameters, while the performance of the system decreases only in the non-realistic case of *H* = *k*_*B*_ *T*. This indicates that the electron-phonon interactions have no influence on the main thermoelectric parameters of the SET related to the electronic conduction. This robustness to energy level broadening is understandable from the expressions of tunnel transfer rates given in (9): as the Fermi-Dirac distribution *f*_*L*_(*ε*) and the density of states *ρ*_*L*_(*ε*) are multiplied by the spectral function *A*(*ε*), we can consider that *f*_*L*_ and *ρ*_*L*_ are constant within the energy scale of a small energy-level broadening. The tunnel transfer rates are then coinciding with that in the case of discrete energy levels.

As expected, the electronic conductance exhibits peaks when the Fermi level of the contact is nearly aligned with the energy level, i.e. when ${E}_{{F}_{L}}\approx {\mu }_{n}$, with a shift due to a self-consistent effect at finite temperature^50^. Besides, the Seebeck coefficient *α* decreases linearly when increasing the gate bias, and is equal to zero when ${E}_{{F}_{L}}={\mu }_{n}$. These results were actually visible in Fig. 4, where a different normalization was used to focus on the linearity of *α* in the mV range of *V*_*DS*_. However, at larger scale we can clearly see here that between two energy levels, *α* switches abruptly from negative to positive values, due to the transition from first (*n* = 1) to second (*n* = 2) level in the QD. During this transition, the gate bias for which the Seebeck coefficient reaches zero depends on the broadening. Indeed, due to the dissymmetry between peaks of conductance, as seen on Fig. 5a, a higher broadening leads to the vanishing of Seebeck coefficient for a lower gate voltage, as observed in Fig. 5b.We will see that it has a consequence on the thermal conductance.

Finally, the power factor (${\alpha }^{2}{G}_{e}$) shows a high peak on one side of the energy levels and a smaller residual peak on the other side. The peaks are actually located near the *G*_*e*_ peaks shifted toward the highest *α*. The first peak reaches a value around 20 aW.K^−2^. This value is quite small, due to the weak value of the conductance achievable in a SET which is below 1 nS. However, it should be reminded that it is achieved for a 4.4-nm QD and it corresponds actually to 1.3 W.m^−2^.K^−2^. That means that a *μ*m^2^ wide SET matrix could be used to supply ultra-low power electronic systems consuming few *μ*W even using low temperature gradients.

### Non-linearity in the relationship between heat and electric currents

**Figure 6:**
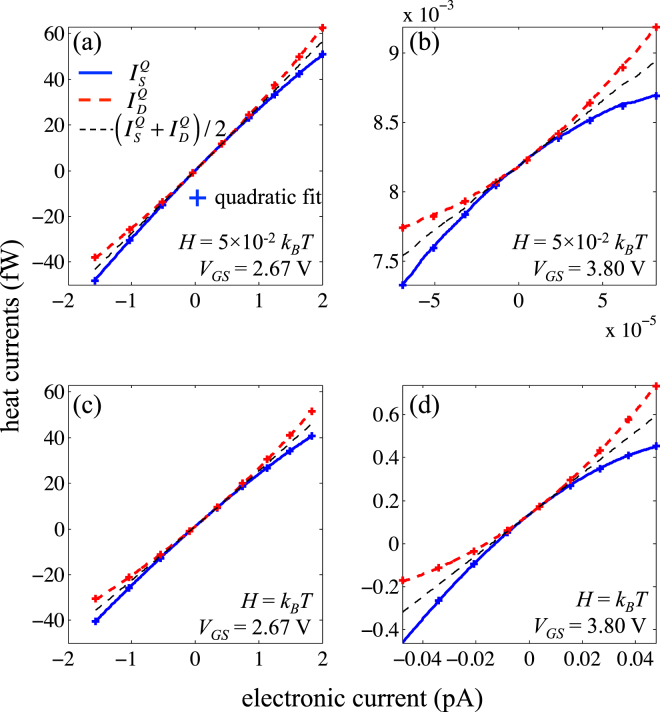
Heat currents ${I}_{S}^{Q}$, ${I}_{D}^{Q}$ and their average as a function of the electronic current *I* for two energy broadenings and two gate voltages, at *T* = 100 K and Δ*T* = 5 K. The crosses represent the quadratic fit for both heat currents.

The heat currents at the hot side ${I}_{S}^{Q}$ and cold side ${I}_{D}^{Q}$ are plotted in Fig. 6 as functions of the electronic current *I* for realistic and un-realistic broadening of energy levels at two different gate voltages. At the first maximum of power factor in Fig. 5c, i.e. *V*_*GS*_ = 2.67 V, both heat and electronic currents are slightly lower in absolute value in the case of a wide broadening *H* = *k*_*B*_ *T* (Fig. 6a and c). It is due to the fact that a part of electrons are no longer in the energy range of high transmission. However, far from the energy levels, i.e. for ${V}_{GS}=3.80$ V (Fig. 6b and d), currents are of course much smaller but they increase significantly in the case of a wide broadening. This can be easily understood by the fact that a wide broadening tends to “spread” the current far from the discrete energy levels, which enhances the opportunities of transmission.

**Figure 7:**
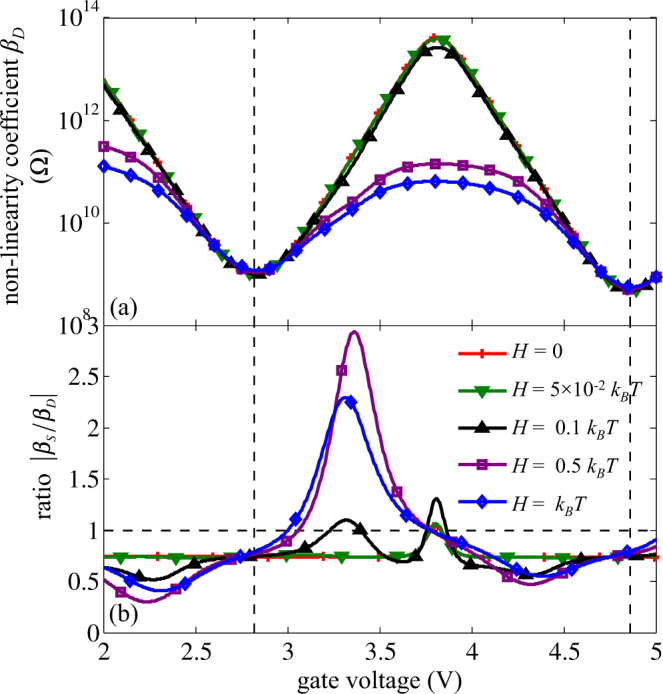
(**a**) Non-linearity coefficient *β*_*D*_ for heat current ${I}_{D}^{Q}$ and (**b**) the ratio |*β*_*D*_/*β*_*S*_| as a function of gate voltage at *T* = 100 K and Δ*T* = 5 K. The vertical lines correspond to the maximum of conductance peaks in Fig. 5a.

Inconsistently with Equation (14), the terminal heat currents ${I}_{S}^{Q}$ and ${I}_{D}^{Q}$ are not linear with respect to the electric current *I*. We have checked that the difference ${I}_{S}^{Q}-{I}_{D}^{Q}$ perfectly fits the dissipated electric power *P*_*d*_ = *V*_*DS*_ × *I* (not shown). This difference between ${I}_{S}^{Q}$ and ${I}_{D}^{Q}$ and their quadratic form is a consequence of a supplementary thermal flux created by Joule effect in the QD i.e. ${I}_{S}^{Q}-{I}_{D}^{Q}={P}_{d}={I}^{2}/{G}_{e}$. The formula (14) should be modified at high bias by adding a non-linearity coefficient *β*, the heat current taking the form of^1^ ${I}_{S}^{Q}={\beta }_{S}{I}^{2}+\alpha TI+K{\rm{\Delta }}T$. A similar expression holds for the heat current at drain side. A quadratic fit of the ${I}_{D}^{Q}-I$ curve (symbols in (Fig. 6) leads to the non-linearity coefficients *β*_*D*_ shown in Fig. 7a. As expected, the evolution of *β*_*D*_ − *β*_*S*_ corresponds to the inverse of the conductance *G*_*e*_ shown in Fig. 5a and it is related to the transition from electronic transport through the first level to electronic transport through the second one. Besides, energy broadening has no significant effect near the conductance peak. If the quadratic approach is efficient here, Whitney has shown that the TE response in the simplest case of a point contact at pinch off presents a rich nonlinear behaviour, where higher-order terms are even more important^34^.

Another interest of our numerical approach is its ability to capture the asymmetric diffusion of the thermal flux induced by Joule heating. Indeed, in standard thermoelectric device, Joule heating, quantified here by ${I}_{S}^{Q}-{I}_{D}^{Q}={I}^{2}({\beta }_{D}-{\beta }_{S})$, is assumed to diffuse symmetrically into the 2 external contacts, thus we would expect to obtain −*β*_*S*_ = *β*_*D*_ = 1/2*G*_*e*_^67^ at least at the conductance peaks. In Fig. 7b, the evolution of the ratio |*β*_*S*_/*β*_*D*_| is plotted. Even without energy broadening the system is asymmetric and $|{\beta }_{S}| < |{\beta }_{D}|$ around the conductance peaks, i.e. the Joule heating is mainly dissipated through the drain. The ratio |*β*_*S*_/*β*_*D*_| = 0.73 corresponds to the ratio of the ohmic resistance (evaluated as the ratio of potential difference to electric current – not shown) between source/dot and dot/drain. One can notice that this ratio lower than 1 is detrimental to the TE efficiency of the SET as it leads to higher thermal flux provided by the hot reservoirs at a given output power.

Moreover, this heat transfer dissymmetry can be tuned by the gate bias even in the case of realistic energy level broadening. Then, the electronic contribution to the thermal flux between source and drain contacts can be modulated. This effect in a SET could be used as a gate controlled multiplexing of the thermal flux.

### Inter-level transitions and figure of merit *ZT*

To estimate the potential of the SET as a thermoelectric device via the figure of merit $ZT={G}_{e}{\alpha }^{2}T/K$, we have to investigate the electronic contribution to the thermal conductance *K*, extracted from the heat current ${I}_{S}^{Q}$ taken at *V*_*oc*_.

**Figure 8:**
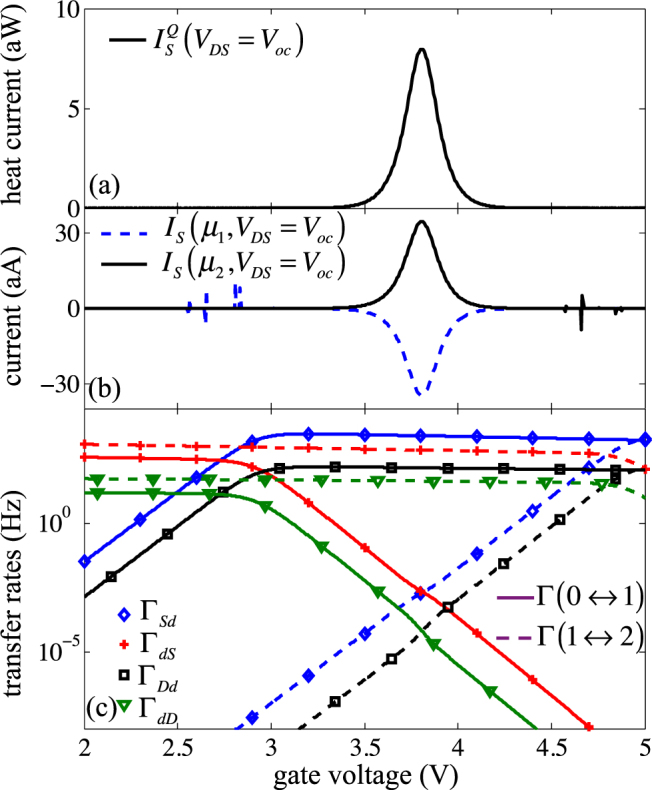
(**a**) Heat current ${I}_{S}^{Q}$ for *V*_*DS*_ = *V*_*oc*_, (**b**) partial electronic currents *I*_*S*_ for *V*_*DS*_ = *V*_*oc*_ considering only the exchange on one level in the QD (1 or 2 electrons) and (**c**) tunnel transfer rates for *V*_*DS*_ = *V*_*oc*_, continuous (dotted) lines for the transfer on the first (second) energy level, as a function of gate voltage, for a discrete case, with *T* = 100 K and Δ*T* = 5 K.

A manifestation of the transition between first and second energy levels can be seen in the heat current at *V*_*oc*_ on the discrete case (Fig. 8a). In the limit of non-broadened energy levels, the electrons that flow through the device can have only discrete energy values, which means that the charge current is always associated with a thermal flux carried by electrons (and vice versa). In a one-level model, we would expect this thermal flux to be null when *I* = 0, all particles carrying the same energy. In the case of a multiple (yet discrete)-level QD, a peak in the heat current – gate voltage characteristics appears even if *I* = 0 (at *V*_*DS*_ = *Voc*), as shown in Fig. 8a. Actually, the center of the peak is located where Γ_*Sd*_(1) = Γ_*dS*_(1) and Γ_*Dd*_(1) = Γ_*dD*_(1) (Fig. 8c), which corresponds to the situation where if one electron is in the QD, the probability for this electron to exit the QD is equal to the probability for another electron to enter. As a consequence, the contributions of both energy levels *I*(*μ*_1_) and *I*(*μ*_2_) to the electronic current are finite, which induces a peak of heat current.

**Figure 9:**
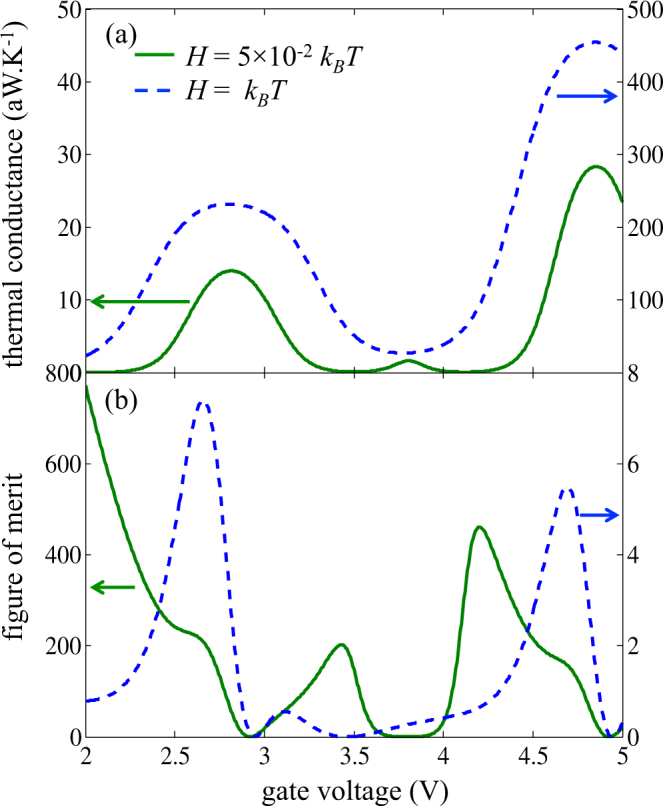
(**a**) Thermal conductance and (**b**) figure of merit ZT as a function of gate bias for two different energy broadenings.

On Fig. 9a, this peak at *V*_*GS*_ = 3.8 V is still visible on the thermal conductance $K={({I}_{S}^{Q}/{\rm{\Delta }}T)}_{{V}_{DS}={V}_{oc}}$ for the realistic case *H* = 0.05*k*_*B*_ *T*, even though it is small compared to the two other peaks centered on the broadened energy levels. This small peak is fully suppressed for *H* = *k*_*B*_ *T*, as the thermal conductance never reaches zero. Regarding the figures of merit plotted in Fig. 9b, a high value of 800 is reached around *V*_*GS*_ = 2 V for a weak but realistic broadening *H* = 0.05*k*_*B*_ *T*, but the power factor is very low for such gate voltage (see Fig. 5c). At the peaks of power factor, the figure of merit is almost 200. The broadening *H* = *k*_*B*_ *T* reduces the figure of merit down to 6, with peaks corresponding to that of the power factor – gate voltage characteristics (Fig. 5c).

One must keep in mind, however, that in this study the contribution to the thermal flux due to lattice vibrations (phonons) is not included. A rough estimation of the thermal conductance of silicon dioxide (≈1 W/K/m) between the contacts would lead to a phonon thermal conductance in the order of nW/K, to be compared with some aW/K for the electronic contribution. It has been shown theoretically and experimentally that by nanostructuring semiconductors it is possible to drastically reduce the lattice contribution to the thermal conductance, as demonstrated for instance in silicon nanowires with different doping levels^13^. However, we may expect this lattice contribution to be dominant in real devices, leading to significant reduction of the absolute values of both *ZT* and thermoelectric efficiency.

### Electric power vs. efficiency

The figure of merit *ZT* is an estimator of thermoelectric conversion efficiency in linear regime. To investigate further the TE properties of the SET even far-from-equilibrium, the electric power and the efficiency have been directly computed. Using the sign convention of Fig. 1, the electric power delivered by the SET in the generator operating regime is *P* = −*I* × *V*_*DS*_ and the thermoelectric efficiency is given by $\eta =P/{I}_{S}^{Q}$, ${I}_{S}^{Q}$ being the thermal flux delivered by the source contact which is here the hot reservoir.

**Figure 10:**
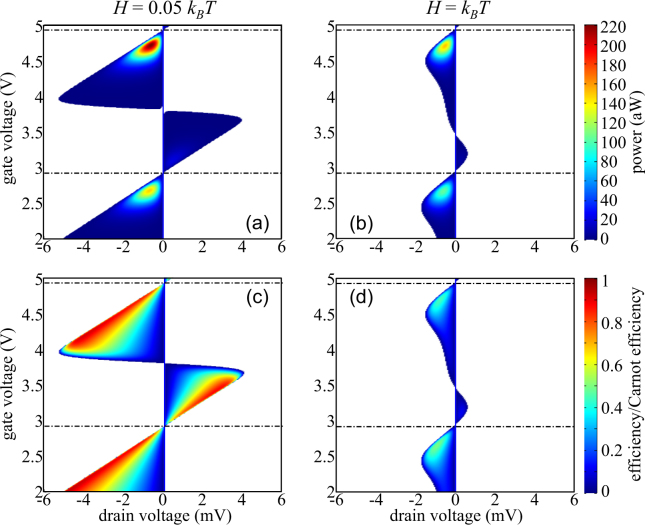
Cartography of (**a**), (**b**) the generated electric power P and (**c**), (**d**) the efficiency *η* normalized by the Carnot efficiency *η*_*c*_, as a function of both the gate (*V*_*GS*_) and drain biases (*V*_*DS*_), for two different energy level broadening and Δ*T* = 5 K in the SET-based thermoelectric generator.

The electric power generated for an applied temperature gradient Δ*T* = 5 K at a temperature *T* = 100 K and for broadenings *H* = 0.05*k*_*B*_ *T* and *H* = *k*_*B*_ *T* are displayed in Fig. 10a and b, respectively. The two maximum of power correspond to the peaks of power factor shown in Fig. 5c. They occur at *V*_*GS*_ = 2.67 V and 4.71 V, respectively, and for *V*_*DS*_ = *V*_*oc*_/2. Again, the low maximum absolute value of the delivered electric power (0.22 fW for *H* = 0.05*k*_*B*_ *T*) must be put into perspective with the nano-size of the QD. This power corresponds to a value of 10 Wm^−2^ which is only 1 order of magnitude lower than for a photo-voltaic system while the temperature gradient considered here is weak, only 5 K.

The respective conversion efficiencies are shown in Fig. 10c and d. When the current is weak, the effect of energy level broadening is limited and the electronic transport is quasi reversible. As a consequence, the Carnot efficiency *η*_*C*_ can be nearly achieved. The maximum of efficiency is thus reached for a current *I* ≈ 0, i.e. in a working regime where the delivered electric power *P* tends to 0, which has very little relevance.

Otherwise, for *V*_*GS*_ = 2.67 V and 4.71 V and for ${V}_{DS}={V}_{oc}\mathrm{/2}$, the efficiency at maximum power reaches 49% (50% is the theoretical maximum value, limited by the impedance matching in electric circuit) of the Carnot efficiency for *H* = 0.05*k*_*B*_ *T*. This ratio decreases down to 38% for *H* = *k*_*B*_ *T*. Similar behaviors have been previously observed in the case of a resonant-tunneling quantum dot^20^. For practical applications, this working regime is actually much more relevant.

## Conclusion

By means of 3D self-consistent simulation, we have explored the thermoelectric properties of a Si-QD based SET in the framework of sequential tunneling regime of transport including realistic collisional broadening of energy levels. The different TE working regimes of the SET i.e. generator, cooler and heat pump have been investigated in terms of electric and heat currents in a large voltage range. Interestingly, it was shown that SETs exhibit *V*_*GS*_-Seebeck voltage characteristics which are material-independent and appear to be weakly dependent on the scattering mechanisms responsible for the energy level broadening inside the dot.

Besides, the transition between the first and the second energy level is shown to lead to a non-linearity of heat currents with respect to electronic currents, as well as a thermal conductance peak, even in the case of discrete energy levels. This non-linearity is asymmetric between the hot and cold side and can be controlled by the gate voltage. The electronic figure of merit *ZT*, i.e. without considering the phonon contribution, reaches a value as high as 200 for realistic energy level broadening. Finally, the SET-based thermoelectric generator was shown to exhibit an efficiency reaching 49% of the Carnot efficiency at maximum power. This maximum power corresponds to a significant power density of 10 W.m^−2^ for a reasonably small temperature gradient of 5 K. Although a full implementation of phonon transport at the nanoscale is mandatory to provide fully quantitative estimation of the efficiency of such thermoelectric generators, their unique TE properties open the way of promising applications in nanoscale metrology.

### Data Availability

The datasets generated and analysed during the current study are available from the corresponding author on reasonable request.
